# Impact of Thermal Control Measures on the Imaging Quality of an Aerial Optoelectronic Sensor

**DOI:** 10.3390/s19122753

**Published:** 2019-06-19

**Authors:** Fuhe Liu, Zhifeng Cheng, Ping Jia, Bao Zhang, Xiaofeng Liu, Rizha Hu

**Affiliations:** 1 Key Laboratory of Airborne Optical Imaging and Measurement, Changchun Institute of Optics, Fine Mechanics and Physics, Chinese Academy of Sciences, Changchun 130033, China; liusong0804@126.com (F.L.); Jiap@ciomp.ac.cn (P.J.); hcciomp@sina.com (B.Z.); liuxiaofeng@ciomp.ac.cn (X.L.); hitabc2007@sina.com (R.H.); 2 University of Chinese Academy of Sciences, Beijing 100049, China

**Keywords:** remote sensor, image analysis, temperature, thermal effects

## Abstract

The image resolution is the most important performance parameter for an aerial optoelectronic sensor. Existing thermal control methods cannot eliminate the sensor’s temperature gradient, making the image resolution difficult to further improve. This article analyzes the different impacts of temperature changes on the imaging resolution and proposes modifications. Firstly, the sensor was subjected to thermo-optical simulation by means of finite element analysis, and the different impacts of temperature changes on the imaging quality were analyzed. According to the simulation results, an active thermal control method is suggested to enhance the temperature uniformity of the sensor. Considering the impacts of active and passive thermal control measures, thermal optical analysis was carried out to predict the performance of the sensor. The results of the analysis show that the imaging quality of the sensor has been significantly improved. The experimental results show that the image resolution of the optoelectronic sensor improved from 47 to 59 lp/mm, which demonstrates that the sensor can produce a high image quality in a low-temperature environment.

## 1. Introduction

As an important means of information acquisition, aerial optoelectronic sensing technology has the advantages of flexibility and real-time performance. It is widely used in fields such as geographic mapping, national defense security, and film and television aerial photography [1,2]. The imaging resolution determines the ability to distinguish distant objects, which is the most important performance parameter for an aerial optoelectronic sensor. Such a sensor needs to detect, recognize, and positively identify targets with pinpoint precision in, for example, emergency rescues and forest fire prevention. In aerial photography, it is necessary to clearly distinguish scenes on the ground. The higher the resolution, the smaller a target can be, and still be able to be successfully identified. There is a direct correlation between the imaging quality and the temperature uniformity of the aerial optoelectronic sensors [3,4,5]. However, existing thermal control methods cannot eliminate the sensor’s temperature gradient, making the image resolution difficult to further improve. Due to the complexity of the aerial environment, the temperature of the sensors changes greatly during the execution of the mission, thus causing serious damage to the imaging resolution. In order to ensure that the imaging device outputs a clear image, thermal control measures must be taken to compensate for the temperature of the optics.

In order to obtain high-resolution images, the thermal control technique has been widely used in spaceborne remote sensors for the past few years. This technology is already widely used in space engineering [6,7,8,9]. The present thermal control research on aerial optoelectronic sensors usually adopts experience acquired in space engineering. Fan, from China, built a finite element model of an aerial sensor, and the heat loss rate was analyzed based on the working atmosphere [10]. A study on the thermal design of a transmission optical remote sensor was conducted by Li [11]. However, the aerial environment was more complex than that in space. There is an intense convective heat transfer between an atmosphere and a sensor in an aerial environment, which makes the sensor’s temperature change greatly. Due to different external thermal conditions, the aerial thermal control technique has its own unique characteristics [12].

Rapid changes in temperature from the ground to the upper air can cause complex heat exchange through conduction, convection, and radiation [13]. As a result, not only are the size and optical properties of the optical components changed, but the refractive index of the glass material is as well, causing the optical system to defocus and thus produce additional aberrations that severely affect the imaging resolution of the optical system [14,15]. Therefore, the use of thermal control technology to stabilize the temperature level of high-altitude optical remote sensors and eliminate the influence of temperature gradients on imaging quality is key in the development of aerial optoelectronic sensors. This article discusses the phenomena found in thermal experiments, proposes modifications for the thermal control system, and demonstrates the importance of temperature uniformity. The impact of temperature changes on the imaging resolution was analyzed, and the improved thermal control measures were found to meet the requirements of the aerial cameras.

## 2. Experimental Phenomenon of the Aerial Optoelectronic Sensor

The aerial optoelectronic sensor is a visible light camera that adopts a Cassegrain optical system. The focal length of the camera is 1200 mm, which is easily affected by thermal conditions. In order to distinguish a target 20 km away, a sensor’s image resolution must be more than 55 lp/mm. The objective of a thermal control method is to ensure that the camera’s resolution at −30 °C is higher than 55 lp/mm. The camera works in a high-altitude aerial environment, so an external cold environment will significantly affect the temperature distribution of the camera’s internal optical system. A passive thermal control design was carried out based on the thermal conditions and structural characteristics of the camera. Passive thermal control measures, such as thermal isolation, were used to extend the thermal time constant of the system and reduce the sensitivity of the optical system to the external thermal environment.

**Figure 1:**
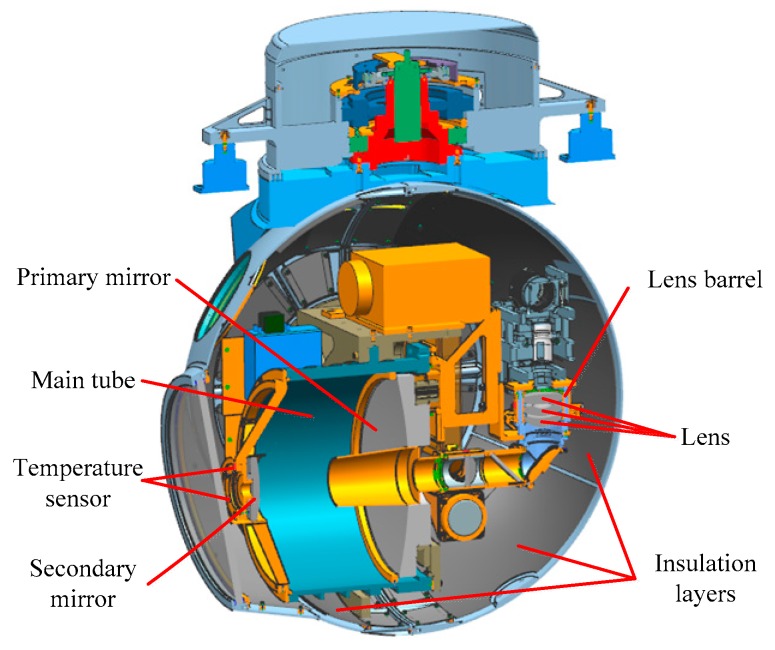
Original thermal control design of the optoelectronic sensor.

In order to reduce the thermal deformation in the optical system, SiC and 4J32 were selected as the material of the mirrors and the supporting structure, respectively, for their similar linear expansion coefficient. The lens could adopt materials such as NBK and NSF with a low linear expansion coefficient, NBK and NSF are common materials used in the field of engineering optics, and the lens barrel material was TC4, which can significantly reduce the thermal stress between the optical component and the mechanical component. The surface of the mechanical components was black, anodized to facilitate a uniform temperature throughout the assembly, and the inner surface of the spherical shell was entirely covered with insulation layers. As shown in Figure 1, the temperature sensors were attached to the primary mirror, the secondary mirror, and the lens barrels.

**Figure 2:**
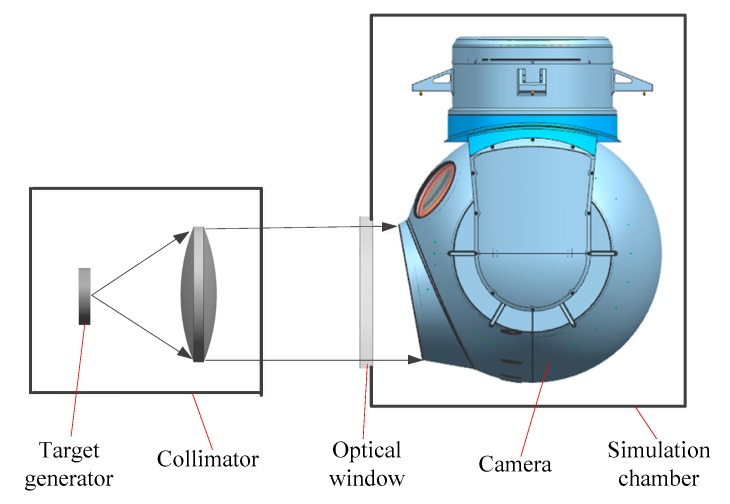
Schematic of the ground resolution test.

Taking pictures of the identification plate is important for testing the sensor resolution in the lab. The identification plate was comprised of black and white stripes, where the width of each stripe was between 10 μm and 40 μm. The sensor’s visual resolution was calculated by identifying the minimum width of the stripe. According to Figure 2, the thermal experimental device mainly comprised a target generator, a collimator, a simulation chamber, and a camera. The test steps were as follows: The initial temperature of the camera was set to 20 °C. The camera was then put into a −30 °C simulation chamber, in which the camera imaged the target projected by the outer collimator. The camera cooled due to the influence of the ambient temperature. Table 1 shows the measured temperature after 1 h. It can be seen that the temperature obviously declined, and the optical system produced an axial temperature gradient. The temperature of the secondary mirror is the lowest, as it is closer to the window, which will generate a relatively strong radiation heat exchange with the external environment.

**Figure 3:**
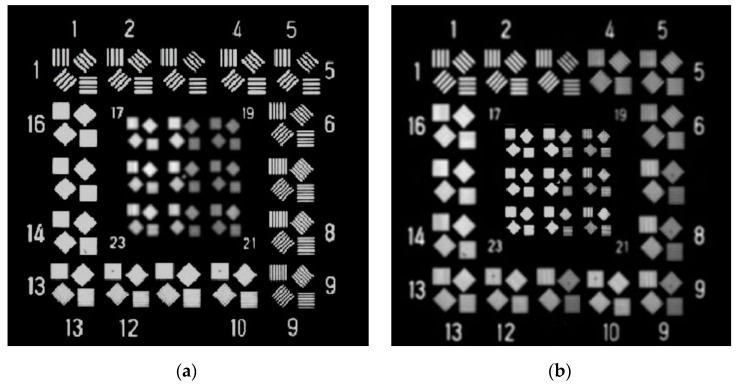
Images obtained at different temperatures: (**a**) at the initial temperature; (**b**) at a cold temperature after 1 h.

It was found that a low temperature environment could degrade the imaging resolution of the camera. The two images in Figure 3 were taken by the camera at different temperatures for the same target. Figure 3a is an image obtained at 20 °C. The smallest resolvable stripe is the ninth group with a resolution of 66 lp/mm. The image in Figure 3b was taken after 1 h, and the minimum resolvable stripe is the third group, with a resolution of 47 lp/mm. The experimental results show that the imaging resolution under the existing passive thermal control does not meet the requirement, which indicates that the existing passive thermal control measures should be modified and redesigned.

## 3. Analysis of the Thermal Imaging Test

In the low-temperature environment, the imaging quality of the optoelectronic sensor descended significantly. This phenomenon was caused by the uneven temperature of the optical system.

### 3.1. Impacts of Temperature Level and Temperature Gradient on Imaging Quality

**Figure 4:**
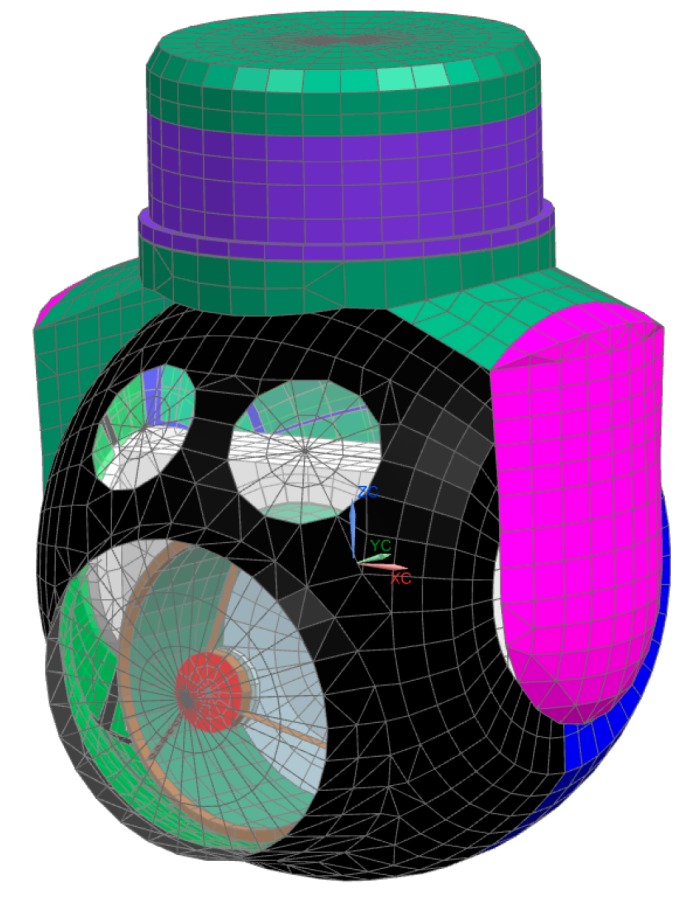
Model of the optoelectronic sensor for thermal analysis.

Analysis of the temperature effects on imaging quality requires a variety of disciplines in mechanics, heat transfer theory, and optics. For a transflective optical imaging system, changes in temperature affect the shape accuracy of the mirrors and the refractive index of the lens. Although the mechanical support structure does not participate in imaging, its thermal stress can be transmitted to the optical component and, at the same time, affects the position and shape of the optical components. All these factors will affect the imaging quality. In order to study the impacts of these factors, finite element analysis should be conducted on the optical system using thermal optics software. The analysis procedure is as follows: (1) Simplify the 3D model, divide the gridding, and build the finite element model of the sensor, as shown in Figure 4; (2) select the temperature level and temperature gradient as the loading conditions, and calculate the deformation of each node under the constraint conditions; (3) import the displacements of the nodes on the optical surface and refractive index of the lens into the optical model; and (4) analyze the effects of these factors on the imaging quality.

During the simulation process, a temperature difference of 30 °C and axial temperature gradient of 30 °C were adopted. While the structural analysis was being completed, the displacement of the mirror surface nodes was put into the software for further optical simulation. Table 2 shows the optical aberrations caused by the temperature level and the temperature gradient. Aberrations can describe the performance of the optoelectronic sensor. The larger the aberration, the worse the imaging resolution. In Table 2, SA, TCO, TAS, and SAS are the main aberrations of the optical system, and they represent the spherical aberration, the coma, the meridional image power, and the sagittal image power, respectively. In Table 2, each row stands for an optical surface. The first two rows are the primary and secondary mirrors, the last 10 rows are the surface of the lens, and the last row is the sum of the aberrations of all surfaces. 

Compared to the aberrations of the primary mirror and the second mirror, the lens aberrations are so small that they can be neglected in the imaging process. This indicates that the mirrors are more sensitive to temperature changes. It can be concluded from Table 2 that the aberrations were mainly distributed in the spherical aberration and the coma. Both the temperature level and the temperature gradient will bring a large spherical aberration to the system; the difference is that the temperature gradient will significantly increase the system’s coma. There is an auto-focusing mechanism in this camera, and the spherical aberration caused by the temperature can be compensated for. Since the system cannot compensate for the coma, the temperature gradient can affect the image quality of the camera. 

### 3.2. Optical Analysis of the Sensor Based on the Present Control Measures

**Figure 5:**
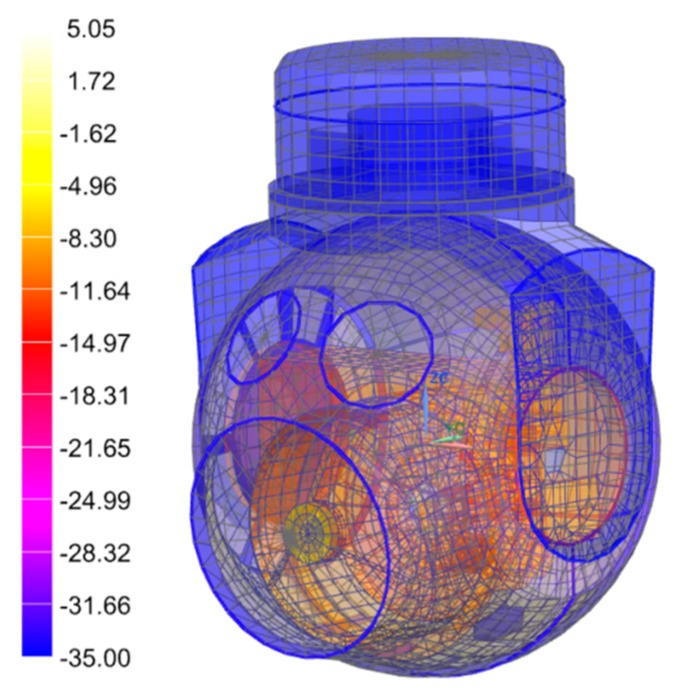
Analysis of temperature distribution after 1 h.

The optical thermal analysis was conducted based on the present thermal control of the sensor. The heat transfer, convection, and radiation conditions were considered during the simulation process. The distribution of temperature in the sensor is shown in Figure 5. According to the results, the distribution of temperature was not uniform when the camera was exposed to a cold environment for an hour. The secondary mirror was the coldest; the temperature was −14.2 °C. The temperature gradient between the primary mirror and the secondary mirror was 6.2 °C. The first three aberrations of the camera are shown in Table 3, in which the spherical aberration and coma are the most important aberrations. The aberrations are mainly in the mirrors, while the aberrations of the lens are very small. It is obvious that the primary and secondary mirrors are more temperature-sensitive.

As shown above, it can be concluded that the decrease in temperature caused the contraction of the main tube, reducing the distance between the primary mirror and the secondary mirror, and thus causing the spherical aberration in the optical system. The temperature gradient led to the thermal stress in the main tube, and the surface figure of the primary mirror and secondary mirror was affected by the stress, thus causing the spherical aberration and coma in the camera. The spherical aberration was improved by the focusing mechanism; however, the coma could not be improved by the system itself. The temperature gradient affected the image quality of the camera. Therefore, the decline in the imaging quality in the test above was mainly caused by the temperature gradient.

## 4. Improvement of Thermal Control Measures

### 4.1. Design of the Active Thermal Control Methods

**Figure 6:**
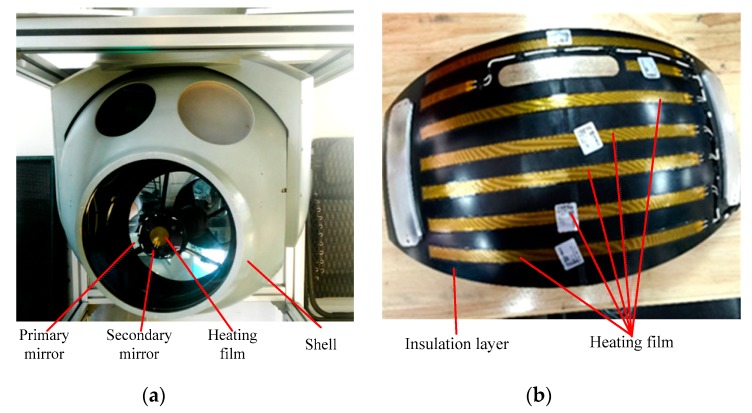
Schematic of the active thermal measure. (**a**) Heating films on the optical systems; (**b**) heating films on the insulation layer.

Based on the analysis above, temperature uniformity is essential for the imaging quality of aerial optoelectronic sensors. While passive thermal control measures work alone, the temperature gradient between the primary and secondary mirrors stays relatively high due to the fast cooling speed of the secondary mirror. This temperature gradient will reduce the imaging quality of the optical system; therefore, active thermal control measures should be added to this camera. Some improvements have been made based on the problems existing in the initial thermal control method. The heating films were attached to the primary mirror, the secondary mirror, and the internal surface of the shell, as shown in Figure 6. The thermal control targets are as follows:

(a) The camera temperature level (the average temperature of the three measurement points of the primary mirror) is 0~20 °C;
(b) temperature gradient between the primary mirror and the secondary mirror is less than 5 °C.

**Figure 7:**
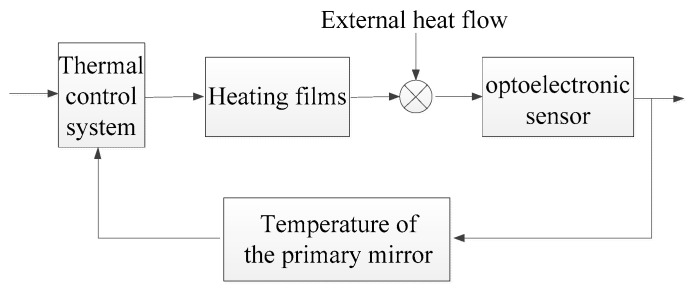
Feedback loop of the thermal control system.

The thermal control strategy is as follows: If the initial temperature of the primary mirror is less than 0 °C, the target temperature is set to 0 °C; if the average temperature of the primary mirror is more than 20 °C, the target temperature is set to 20 °C. The DS18B20 was used to measure the temperature of the sensor, and the accuracy of this detector is 0.1 °C. The DS18B20 is a digital commercial thermal detector that has been used successfully in space remote sensors. The heating films are powered off when the temperature of the secondary mirror is 0.2 °C greater than the target, and the heating films are powered on when the temperature of the secondary mirror is lower than the target. As shown in Figure 7, the thermal control is achieved through a feedback loop, and the temperature of the primary mirror is used as the feedback.

### 4.2. Optical Analysis of the Sensor Based on the Modified Control Measures

The boundary conditions were reset based on the improved thermal control method, while the finite element model was the same as that described in Section 3.2. The thermal analysis results show that the overall temperature level of the camera was significantly improved, and the temperature difference between the primary and secondary mirrors was also significantly reduced. After the thermal optical analysis, the first three orders of aberrations of the system were obtained, as shown in Table 4. With the help of the active thermal control, the spherical aberration of the optical system was improved, and the coma was more obviously reduced. The finite element analysis results show that the combination of active thermal control and passive thermal control can achieve satisfactory thermal control results.

### 4.3. Experimental Verification

**Figure 8:**
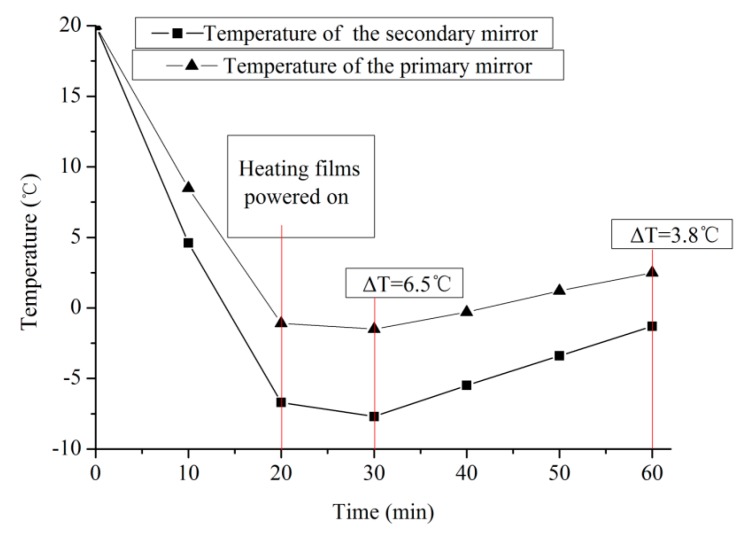
Temperature changes of the primary mirror and the secondary mirror during the test.

Experiments were carried out on the improved camera in accordance with the thermal control scheme, and the imaging resolution of the camera in a low-temperature environment was obtained. The initial temperature of the camera, placed in an environment of −30 °C, was 20 °C. After 20 min, the camera and thermal control system began to work. Figure 8 shows the temperature changes of the mirrors during the test. The temperature gradient of the two mirrors increased rapidly in the first 20 min. According to the thermal control strategy above, the control target for the primary mirror temperature was below 0 °C when the films were powered on. Once the heating films were powered on, the gradient gradually decreased to 3.8 °C. The temperature of the primary mirror was 2.5 °C after 60 min, and the thermal distribution reached the control target. It can be concluded that the accuracy of the modified system was 2.5 °C, and the control time was 1 h. 

**Figure 9:**
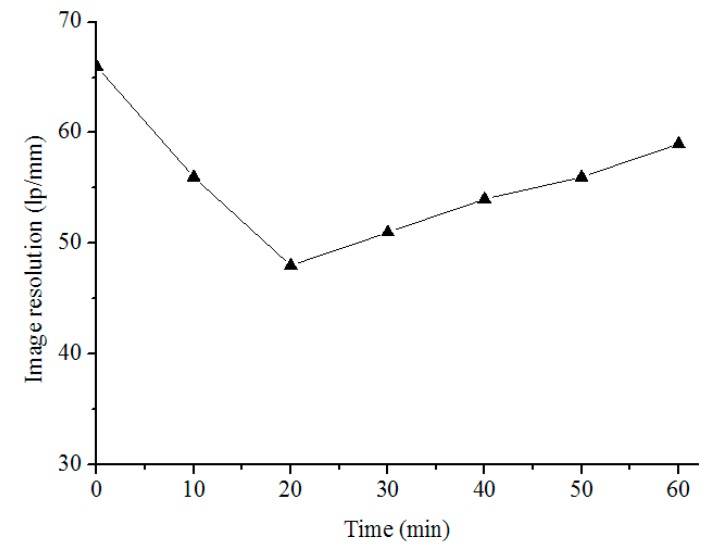
Image resolution of the sensor during the test.

**Figure 10:**
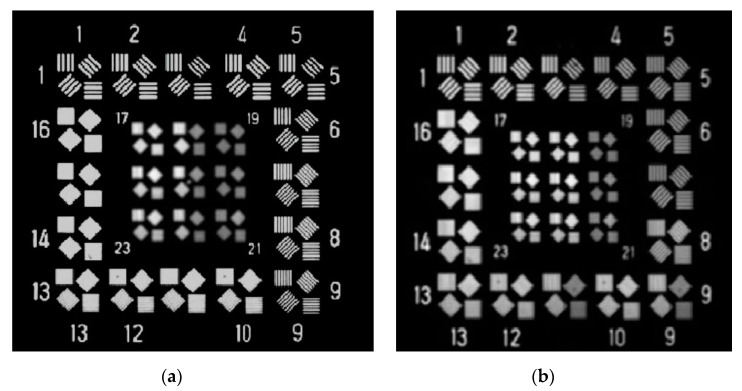
Images obtained at different temperatures: (**a**) at the initial temperature; (**b**) at low temperatures after 1 h.

The resolution during the test and the target image obtained by the sensor are shown in Figure 9 and Figure 10, respectively. The image resolution declined, while the temperature gradient of the two mirrors increased rapidly in the first 20 min. Once the films were powered on, the image resolution improved from 47 lp/mm to 59 lp/mm. It can be seen that the image resolution in Figure 10b is better than that in Figure 3b. With the help of the heating films, the camera could distinguish the seventh group image at a low temperature. It can be seen that the modified thermal control system improved the imaging resolution of the aerial optoelectronic sensor, which proves the effectiveness of the modified thermal control system.

## 5. Conclusions

Thermal control measures are essential for the imaging quality of aerial optoelectronic sensors. This paper analyzed the influence of temperature on the aberration of aerial optoelectronic sensors, verified the importance of temperature uniformity, and improved the imaging resolution by modifying the thermal control method. The experimental results show that the imaging resolution improved from 47 to 59 lp/mm under modified thermal control measures, which met the resolution requirement of the sensor. The research in this paper provides a reference for the thermal control design of high-resolution aerial optoelectronic sensors.

## Figures and Tables

**Table 1:** Temperature under the thermal control.

Sensor Location	Temperature (°C)
Primary mirror	−8.3
Secondary mirror	−14.9
Main tube	−8.5
Lens barrel	−7.6

**Table 2:** Analysis of third-order aberrations.

	SA	TCO	TAS	SAS
(a) Temperature level difference of 20 °C				
1	−3.366	0.936	−0.038	−0.007
2	1.909	−0.451	0.027	0.002
3	0.042	−0.019	0.001	0.003
4	0.017	0.008	0.002	0.001
5	0.048	0.087	0.078	0.036
6	−0.023	−0.036	−0.051	−0.015
7	−0.239	−0.172	−0.037	−0.022
8	0.004	0.013	0.032	0.005
9	−0.004	0.017	−0.009	0.003
10	0.135	0.092	0.023	0.011
11	−0.009	0.021	−0.006	−0.002
12	0.018	−0.015	0.005	0.006
Sum	−1.468	0.481	0.066	0.021
(b) Temperature gradient of 20 °C				
1	−3.985	1.539	−0.361	−0.093
2	2.297	−0.885	0.236	0.003
3	0.061	−0.092	0.043	0.009
4	0.013	0.035	0.027	0.012
5	0.082	0.405	0.056	0.237
6	−0.032	−0.205	−0.576	−0.573
7	−0.121	−0.243	−0.529	−0.162
8	0.019	0.103	0.231	0.006
9	−0.013	0.075	−0.132	0.063
10	0.098	0.274	0.285	0.097
11	−0.008	0.047	−0.139	−0.003
12	0.037	−0.066	0.103	0.035
Sum	−1.552	0.987	−0.756	−0.369

**Table 3:** Third-order aberration analysis in the original thermal control state.

	SA	TCO	TAS	SAS
1	−5.359	1.623	−0.164	−0.021
2	2.891	−0.563	0.035	0.008
3	0.068	−0.024	0.008	0.009
4	0.023	0.012	0.006	0.005
5	0.057	0.095	0.086	0.053
6	−0.033	−0.041	−0.072	−0.022
7	−0.265	−0.187	−0.043	−0.036
8	0.007	0.027	0.039	0.008
9	−0.015	0.031	−0.024	0.011
10	0.215	0.125	0.037	0.023
11	−0.015	0.033	−0.022	−0.018
12	0.021	−0.032	0.021	0.031
Sum	−2.405	1.123	−0.093	0.053

**Table 4:** Third-order aberration analysis based on the modified thermal control measures.

	SA	TCO	TAS	SAS
1	−2.085	0.351	−0.159	−0.016
2	1.093	−0.216	0.047	0.007
3	0.057	−0.022	0.009	0.017
4	0.019	0.011	0.005	0.013
5	0.058	0.088	0.075	0.028
6	−0.029	−0.035	−0.098	−0.015
7	−0.158	−0.101	−0.040	−0.041
8	0.009	0.032	0.035	0.009
9	−0.016	0.017	−0.033	0.018
10	0.117	0.102	0.052	0.036
11	−0.012	0.029	−0.078	−0.025
12	0.018	−0.018	0.009	0.029
Sum	−0.927	0.238	−0.136	0.061
